# A Novel Null Homozygous Mutation Confirms *CACNA2D2* as a Gene Mutated in Epileptic Encephalopathy

**DOI:** 10.1371/journal.pone.0082154

**Published:** 2013-12-16

**Authors:** Tommaso Pippucci, Antonia Parmeggiani, Flavia Palombo, Alessandra Maresca, Andrea Angius, Laura Crisponi, Francesco Cucca, Rocco Liguori, Maria Lucia Valentino, Marco Seri, Valerio Carelli

**Affiliations:** 1 U.O. Genetica Medica, Policlinico Sant’Orsola-Malpighi, University of Bologna, Bologna, Italy; 2 IRCCS Istituto delle Scienze Neurologiche di Bologna, Bologna, Italy; 3 Dipartimento di Scienze Mediche Chirurgiche, University of Bologna, Bologna, Italy; 4 Dipartimento di Scienze Biomediche e Neuromotorie (DIBINEM), University of Bologna, Bologna, Italy; 5 Istituto di Ricerca Genetica e Biomedica, Consiglio Nazionale delle Ricerche, Cagliari, Italy; 6 Center for Advanced Studies, Research, and Development in Sardinia (CRS4), AGCT Program, Parco Scientifico e tecnologico della Sardegna, Pula, Italy; 7 Dipartimento di Scienze Biomediche, University of Sassari, Sassari, Italy; Odense University Hospital, Denmark

## Abstract

Contribution to epileptic encephalopathy (EE) of mutations in *CACNA2D2*, encoding α2δ-2 subunit of Voltage Dependent Calcium Channels, is unclear. To date only one *CACNA2D2* mutation altering channel functionality has been identified in a single family. In the same family, a rare *CELSR3* polymorphism also segregated with disease. Involvement of *CACNA2D2* in EE is therefore not confirmed, while that of *CELSR3* is questionable. In a patient with epilepsy, dyskinesia, cerebellar atrophy, psychomotor delay and dysmorphic features, offspring to consanguineous parents, we performed whole exome sequencing (WES) for homozygosity mapping and mutation detection. WES identified extended autozygosity on chromosome 3, containing two novel homozygous candidate mutations: c.1295delA (p.Asn432fs) in *CACNA2D2* and c.G6407A (p.Gly2136Asp) in *CELSR3*. Gene prioritization pointed to *CACNA2D2* as the most prominent candidate gene. The WES finding in *CACNA2D2* resulted to be statistically significant (p = 0.032), unlike that in *CELSR3*. *CACNA2D2* homozygous c.1295delA essentially abolished α2δ-2 expression. In summary, we identified a novel null *CACNA2D2* mutation associated to a clinical phenotype strikingly similar to the *Cacna2d2* null mouse model. Molecular and statistical analyses together argued in favor of a causal contribution of *CACNA2D2* mutations to EE, while suggested that finding in *CELSR3*, although potentially damaging, is likely incidental.

## Introduction

Epileptic Encephalopathies (EEs) are severe brain disorders in which the seizures and the epileptic activity itself may cause severe psychomotor impairment. EEs may arise from the neonatal to the early infantile period as recurrent, prolonged or drug resistant seizures, resulting in devastating permanent global developmental delay with brain atrophy. Occasionally, EEs can be associated to brain lesions or malformations of cortical development [1]. EEs are genetically heterogeneous. Numerous genes, all involved in diverse primary developmental processes of the brain, have been already identified and their number and that of the associated clinical spectrum is expanding continuously [1]. Among these the so-called “channelopathies”, originating from defects in genes coding for neuronal ion channels, play a prominent role in monogenic epilepsies, among which EEs [2].

Mutations in *CACNA1A* (MIM 601011), [3] encoding the transmembrane pore-forming subunit Ca_V_2.1 of Voltage Dependent Calcium Channels (VDCCs), [4] have been associated to the peculiar phenotypic combination of absence epilepsy and cerebellar ataxia. Auxiliary regulatory subunits α2δ, β and γ associate with the pore forming α1 subunit and modulate its function [5], [6]. Several mouse models, all characterized by homozygous mutations in one of the genes encoding VDCC subunits, share similar phenotypes including cerebellar ataxia, paroxysmal dyskinesia and seizures similar to those of absence epilepsy as well as other forms of generalized epilepsy. Among these the *ducky* mutant mice, which carry null alleles in *Cacna2d2*, represent a model for absence epilepsy characterized by behavioral arrest synchronous with spike-wave discharges and cerebellar ataxia [7], [8]. α2δ is encoded by a single gene and is post-translationally modified to form two proteins, δ and α2: the δ piece, a single-pass trans-membrane portion, anchors the α2 protein to the membrane [9]. It acts mainly by enhancing the trafficking or reducing the turnover of the channel complex in the plasma membrane [8], [10]. The α2δ-2 subunit is involved in the composition of a variety of different VDCCs, but it contributes mainly to Cav2.1/β4 (P-type current) in central synapses [9].

Recently, a homozygous *CACNA2D2* (Calcium Channel, Voltage Dependent, α2δ subunit 2; MIM 607082) mutation was identified in a family with 3 siblings, offspring to consanguineous parents, who presented with early-onset epileptic encephalopathy and global developmental delay. This mutation, a probably pathogenic missense p.Leu1040Pro substitution, affected a highly conserved residue and was shown to cause dysfunction of α2δ-2, resulting in reduced current density and slow inactivation in neuronal calcium channels (**Table 1**) [11]. Another possibly detrimental rare p.Met2630Ile polymorphism (rs149614835) in *CELSR3* (Cadherin EGF LAG 7-pass G-type receptor 3; MIM 604264) (**Table 1**) segregated with the disease phenotype in the same family. These findings supported the hypothesis that defective α2δ-2 may underlie the epileptic phenotype. However, this observation was not confirmed in independent patients and a role in disease pathogenesis of the concomitant *CELSR3* variant could not be ruled out [11]. Here we report on abolished expression of α2δ-2 in a patient, offspring to consanguineous parents characterized by the association of epilepsy with also absence seizures, dyskinetic movements and cerebellar atrophy, a clinical picture closely resembling the phenotype displayed by the ducky mouse.

**Table 1:** Features of the CACNA2D2 and CELSR3 mutations.

	Present work		Edvardson et al., 2013 (11)	
Gene	*CACNA2D2*	*CELSR3*	*CACNA2D2*	*CELSR3*
**Frequency**	Novel	Novel	Novel	<0.001 (rs149614835)
**Genomic position**	chr3∶50416390	chr3∶48687978	chr3∶50402595	chr3∶48682550
**cDNA change**	c.1295delA	c.341G>A	c.3119A>G	c.7890G>A
**Protein change**	p.N432fs*	p.G114D	p.L1040P	p.M2630I
**GERP score** §	–	5.07	4.76	5.24
**Pathogenicity prediction**	–	Tolerated (SIFT) Damaging (MT) Probably Damaging (Polyphen2)	Damaging (SIFT, MT)	Damaging (SIFT, MT)

^§^ GERP (Genomic Evolutionary Rate Profiling) is a measure of the nucleotide evolutionary conservation. It ranges from −12.3 to 6.17, with 6.17 being the most conserved [30].

## Materials and Methods

### Case Report

The proband is an Italian 9-years-old boy, offspring to first cousins, with an older and healthy sister. We followed this patient since the age of 2 years. He was born at 40 weeks after an uneventful pregnancy and delivery. During the first months of life, the child presented hypertonus and eye rolling movements. Afterwards, a severe delay of psychomotor development became apparent, characterized by legs hypertonia, axial hypotonia, dyskinetic movements and myoclonic jerks of the arms and the head, no eye contact, and uncoordinated eye movements. The first epileptic seizure occurred at 5 months without fever, characterized by salivation, loss of contact and clonic jerks on the left side of the body. The first seizure lasted 20 minutes and the interictal EEG showed slow waves on the left occipital regions. Over time, several seizure types occurred, characterized by: a) left eye and head deviation with clonic jerks on the left side of the body; b) generalized hypertonia, loss of contact, uncoordinated movements of eye and limbs, cyanosis, and facial and arms twitching; or c) loss of contact, generalized hypotonia and clonic jerks, and tachycardia or bradychardia. Occasionally, at the end of the ictal event, dyskinetic (particularly choreiform) movements of the limbs appeared, without recovery of consciousness. The seizures, with or without fever, could be prolonged (20–30 minutes) and were frequently followed by sleep. Between seizures, the child always showed head tonic extension, erratic limb movements and tremor. The interictal EEG showed multifocal spikes over the right centro-temporal and the left parieto-occipital regions.

**Figure 1:**
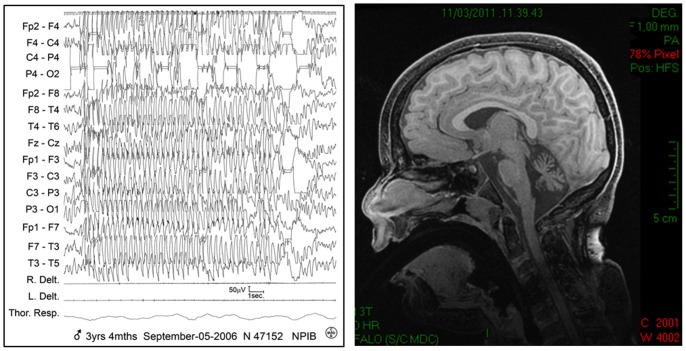
Instrumental findings in the proband. Left panel: EEG recording during wakefulness showing generalized spike-wave complex at 4 Hz lasting thirteen seconds with absence, eye up deviation and eyelid myoclonia. Right panel: Sagittal T1 MRI showing prominent cerebellar atrophy.

At three years of age, absences with eye up deviation and eyelid myoclonus appeared (**Figure 1**). The other seizure types persisted, although reduced in frequency. Seizures were resistant to many drugs (phenobarbital, benzodiazepine, valproic acid, levetiracetam, lamotrigine); absences frequency was improved by ethosuximide. The EEG recordings showed slowing background activity, multifocal, diffuse paroxysmal abnormalities, and a transitory photosensitivity.

Clinical examination showed dysmorphisms(bilateral epicanthus, arched palate, pronounced Cupid’s bow, narrow naris, clinodactyly of the IV and V fingers) and head circumferences between 3 and 10 percentiles. Neurologic examination showed oculo-motor apraxia, strabismus, nystagmus, axial and leg hypertonia, head tonic extension, erratic limb movements, tremor and brisk symmetric reflexes. At the most recent follow-up, it was substantially unchanged, and the child had two types of seizures: very brief absences during wakefulness and tonic-clonic seizures during sleep, which were prevalent on the left side. Laboratory investigations showed hyperglycaemia, and glicosuria. Genetic (high resolution karyotype, analysis for Angelman and Dravet syndromes) and metabolic investigations (urine organic acids, amino acids, isoelectric focusing of transferrins), including those for mitochondrial pathology, were all negative. The ECG and cardiac examination were normal. Brain MRI was remarkable for cerebellar atrophy (**Figure 1**).

### Whole Exome Sequencing for Variant Detection and Autozygosity Mapping

Whole exome DNA from patient’s whole blood was captured using the TruSeq exome enrichment kit (Illumina Inc., San Diego, CA, USA) and sequenced as 100 bp paired-end reads on Illumina HiSeq2000 platform (Illumina Inc., San Diego, CA, USA).

Generated reads have been deposited in the European Nucleotide Archive with accession number PRJEB4676 (http://www.ebi.ac.uk/ena/data/view/PRJEB4676). Reads were checked with FastQC (http://www.bioinformatics.babraham.ac.uk/publications.html) and aligned with BWA [12] to the reference genome hg19. Aligned reads were treated for realignment and base quality score recalibration with GATK, [13] and for duplicate removal with PicardTools (http://picartools.sourceforge.net). Alignment statistics were collected by SAMtools [14] and GATK. Coverage statistics over the targeted regions were calculated with GATK. Variant calling and filtering by quality were performed by GATK. Variants passing quality filters were annotated separately against NCBI RefGene (http://www.ncbi.nlm.nih.gov) and UCSC KnownGene (http://genome.ucsc.edu).

WES genotypes of polymorphic sites present in dbSNP135 (http://www.ncbi.nlm.nih.gov/snp/) were retrieved to create a genetic map consisting of 1673111 sites in the targeted exome, in order to perform autozygosity mapping in the proband as described elsewhere [15].

### Gene Prioritization

To enhance prioritization of candidate genes harboring novel, probably detrimental variants detected by WES, we used Exomiser (http://www.sanger.ac.uk/resources/databases/exomiser/) [16], an on-line tool that functionally annotates and prioritizes mutated genes using criteria as variant frequency, predicted pathogenicity, inheritance pattern and model organism phenotype data. Scores are based on Mutation Taster [17], SIFT [18] and Polyphen2 [19] for predicted pathogenicity of mutations and on Mouse PhenoDigm [20] for phenotypical overlap with the animal model. We adopted the following criteria: 1) autosomal recessive model; 2) phenotypic classification according to the HPO (Human Phenotype Ontology) terms (http://www.human-phenotype-ontology.org): a) epileptiform EEG discharges, b) cerebellar atrophy and c) dyskinesia; 3) removal of variants with allele frequency >1% and predicted as non-pathogenic. To calculate the probability that a mutation falling in a candidate gene is truly associated with disease, we used the on-line tool Exome Power Calculator [21] (exomepower.ssg.uab.edu/) to obtain p-values as measure of statistical significance of the WES findings. Exome Power Calculator provides a simple statistical framework to guide quantitative data analysis by setting the following parameters: 1) Ps, sequence sensitivity: the probability that a variant in the targeted regions is correctly called; 2) m, per individual number of candidate variants; 3) w, relative gene length (ratio of the protein length to the genomic average); 4) n, sample size (number of unrelated patients sequenced); 5) M, total number of genes in the mutational target; 6) a, significance level of the test after Bonferroni correction; 7) o, observed value for the statistic; 8) T, underlying genetic model (dominant/recessive/additive). We assumed Ps = 1 (100% sensitivity), a = 0.05, M = 20653 [22], the underlying genetic model Tr. From the Uniprot database (http://www.uniprot.org/), we retrieved the protein product length of *CACNA2D2* (1150 amino acids) and *CELSR3* (3312 amino acids). Based on the average genomic protein length of 447 amino acids [23], we calculated w = 2 for *CACNA2D2* (1150/447) and w = 7 for *CELSR3* (3312/447). We calculated m as the mean individual number of “candidate variants” from our internal exome database (n = 50 exomes). Since we found a loss-of-function mutation in *CACNA2D2*, we calculated m either including all the coding nonsynonymous, canonical splice-site and coding small indels mutations with population frequency <1% (m_all_), or applying a more stringent filter by selecting only loss-of-function (nonsense mutations and coding small frameshift indels with population frequency <1%) mutations (m_lof_). m_all_ resulted to be 584, while m_lof_ 19.

### Muscle Biopsy

Muscle biopsy of the proband was performed by open surgery after informed consent. Four normal muscle biopsies were used as controls. Muscle specimens were frozen in cooled isopentane and stored in liquid nitrogen. Standard staining (H&E, Gomori modified trichrome, Oil Red O, PAS) and histoenzymatic activities (SDH, COX, NADH, ATPase pH 9.4 and 4.3) were performed following standard protocol [24].

### Expression Analysis

Total RNA was extracted from 150 muscle slices (20 µm thick) by TriPure isolation reagent (Roche) and 1 µg of total RNA was reverse transcribed using the Transcriptor First Strand cDNA Synthesis Kit (Roche). *CACNA2D2* expression was evaluated by real time-PCR, using the Universal Probe Library (Roche) system. The analysis has been conducted in triplicate and the *CACNA2D2* concentration has been calculated through a standard curve by absolute quantification and normalized on *TUBB* expression.

Total proteins were extracted from 50 muscle slices (20 µm thick) adding 100 µl of RIPA buffer (50 mM Tris–Cl pH 7.6, 150 mM NaCl, 1% NP-40, 1% NaDOC, 0.1% SDS, 5 mM EDTA) and 100 µl/ml of protease inhibitor cocktail (Roche). The lysates were sonicated and centrifuged at 10000 *g* and the protein content of the supernatant was determined according to Bradford [25]. Proteins (100 µg) were separated by 8% SDS–PAGE and transferred onto a nitrocellulose membrane (Bio-Rad). Monoclonal primary antibodies specific for *CACNA2D2* 65–162 aa (Abnova M12 clone 4E3, 1∶100) and GAPDH (Sigma Aldrich, G8795 clone 71.1, 1∶20000) were visualized using horseradish peroxidase-conjugated secondary antibodies (Invitrogen, 1∶2000). Signals were detected using Immobilon™ Western peroxidase substrate (Merck-Millipore). Densitometry was performed with a LAS-3000 Imaging System (FUJIFILM) and Image J software [26]. The analysis was conducted in triplicate and *CACNA2D2* protein expression was normalized on GAPDH expression in each sample. Anova test was used to evidence significant differences between groups.

This study was approved by the local ethics committee of Polyclinic Sant’Orsola-Malpighi, Bologna, Italy. We obtained written informed consent from both the parents. All clinical investigation was conducted according to the principles expressed in the declaration of Helsinki.

## Results

**Figure 2:**
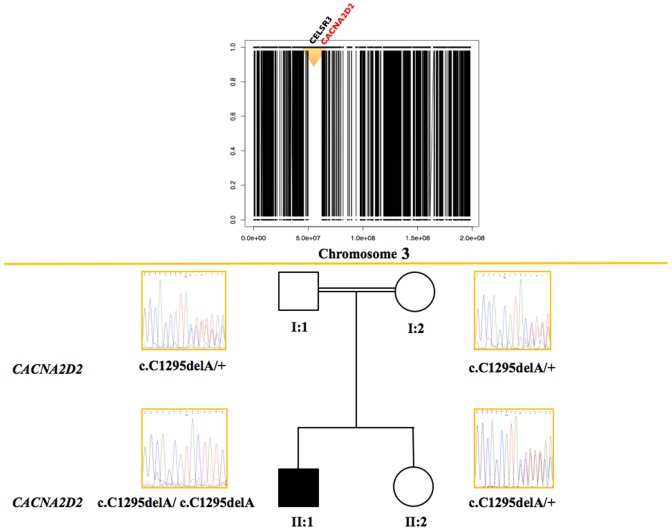
Genetic mapping and segregation of *CACNA2D2* c.C1295delA mutation. Upper panel: barcode plot showing EX-HOM on chromosome 3. If the SNP is heterozygous, y = 0, if it is homozygous y = 1. Black bars represent regions of mixed heterozygous/homozygous SNPs, whilst white bars regions of contiguous homozygous SNPs. Different colors reflect the type of mutation: *CELSR3* has a missense variant (black), *CACNA2D2* has a loss-of-function variant (red). Lower panel: the *CACNA2D2* c.C1295delA mutation segregates with the disease in the nuclear pedigree (parents are 1^st^ cousins).

**Figure 3:**
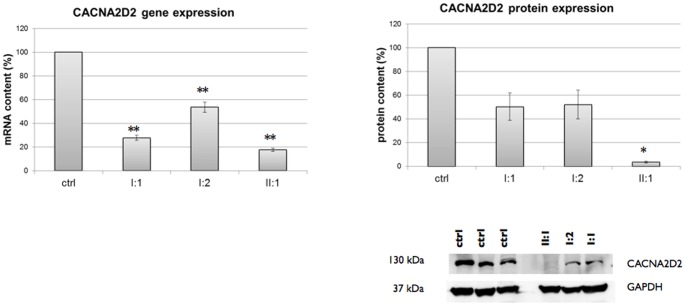
*CACNA2D2* mRNA and protein expression. Left panel: the mRNA content of the proband was evaluated by Real Time-PCR and normalized on that of control individuals (n = 4). *CACNA2D2* mRNA resulted strongly reduced in the proband (II:1, 18%), father (I:1, 28%) and mother (I:2, 54%) compared to controls (Anova test, p<0.001). Data are shown as mean ± standard error of three independent experiments. Right panel: the protein content was evaluated by Western blot and normalized on that of control individuals (n = 3), using GAPDH as reference protein and loading control. Graph shows data obtained by densitometric analysis. The proband (II:1) showed a 3% of protein expression compared to controls (Anova on Ranks test, p = 0.034), whereas both parents (I:1 and I:2) had ∼50% of expression. Data are expressed as mean ± standard error of three independent experiments. Representative western blot is shown.

WES yielded 88.24X mean coverage over the targeted exome with the 80.2% of the exomic positions covered >20X. Due to the recent parental relatedness, we selected the 13 regions >5 megabases (**Table S1**) as the ones with the highest probability to be autozygous and therefore to carry the causative variant among all the *EX-HOM* regions identified in the proband. We selected candidate genes as those harboring variants with reported frequency <1% or no reported frequency, and predicted to alter the protein product (nonsynonymous SNVs, canonical splice-site mutations and InDels). We used Exomiser to prioritize variants according to mutation type, predicted pathogenicity score and association to human or animal model phenotypes. The top-scored variant (**Table S2**) was the c.1295delA (p.Asn432fs) frame-shift deletion in *CACNA2D2* (**Table 1**). This 1-nucleotide deletion received among the highest scores for both pathogenicity (0.95) and phenotype (0.88), the former due to deleteriousness of the frameshift mutation, the latter to the striking similarity between the clinical picture and the *Cacna2d2*-null mouse models phenotypes. This variant lied within the longest proband’s autozygous region (**Table S1 and** **Figure 2**) and was confirmed by Sanger Sequencing as homozygous in the proband and as heterozygous in the healthy sister and in the two parents (**Figure 2**). The p.Asn432fs mutation causes truncation in the the α2δ-2 protein at the level of the α2 piece, which is composed by aminoacid residues from 19 to 1001 [9]. This predicts a protein that lacks anchoring to the membrane and therefore is unable to assemble with the pore forming subunit. In the brain, the protein encoded by *CACNA2D2*, α2δ-2, is mostly abundant in the cerebellum and particularly in Purkinje cells, [27] consistent with the patient’s cerebellar atrophy. The muscle biopsy from the proband, histologically characterized by some variability of fiber size with occasional hypotrophic type II fibers and signs of mitochondrial subsarcolemmal proliferation (data not shown), demonstrated that *CACNA2D2* expression was dramatically reduced compared to controls (**Figure 3**). mRNA quantification showed a ∼80% of reduction of *CACNA2D2* gene expression in the proband compared to control individuals (p<0.001), whereas the father and the mother showed a reduction of ∼70% and ∼50% (p<0.001) respectively (**Figure 3, left panel**). Accordingly, the protein in the proband resulted almost absent (3% of expression, p<0.05), and both the parents presented ∼50% of *CACNA2D2* protein expression (**Figure 3, right panel**).

These findings enforced the hypothesis that dysfunction of α2δ-2 is a cause of EE. However, as in the previous report of a *CACNA2D2* mutation in early onset EE, [11] we noticed the concomitant occurrence of a *CELSR3* variant that segregated with disease in the family (data not shown). This was a nonsynonymous c.6407G>A (p.Gly2136Asp) change, highly conserved and predicted as deleterious by 2/3 predictors (**Table 1**). This reintroduced the question whether *CACNA2D2* and *CELSR3* mutations might act together in causing the epileptic phenotype. In order to evaluate the probability that each of these variants is truly disease-associated and not an incidental WES finding, we calculated the level of statistical significance of finding a *CACNA2D2* or *CELSR3* variant in an individual WES experiment using Exome Power Calculator. Only the occurrence of a recessive loss-of-function mutation in *CACNA2D2* reached statistical significance (p = 0.032) (**Table 2**). Based on molecular and statistical evaluation we concluded that the loss-of-function variant in *CACNA2D2*, leading to abolished expression of the encoded protein, was causative of EE in the proband while the nonsynonymous change in *CELSR3*, although potentially damaging, was likely an incidental finding.

**Table 2:** Calculation of the level of statistical significance of WES findings in candidate genes.

m (n° variants)	*CACNA2D2* (w = 2)	*CELSR3* (w = 7)
**mall (584)**	0,999	1,000
**mlof (19)**	***0,032***	0,109

## Discussion

Our study strengthens the evidence that *CACNA2D2* loss-of-function mutation causes EE. As highlighted by gene prioritization using the Exomiser tool, p.Asn432fs in *CACNA2D2* emerged as the most prominent candidate from the WES performed in the proband. Expression data clearly showed that α2δ-2 was virtually absent in the carrier of the homozygous p.Asn432fs mutation, involving mutations of regulatory VDCC subunits, so far extensively studied only in the animal models, in human epilepsy. The striking similarity between the patient’s and the ducky mouse model phenotypes strongly implicated *CACNA2D2* in the disease pathogenesis. Mouse and human α2δ-2 are 95% identical [7]. In both mice carrying homozygous ducky alleles (du/du and du2J/du2J), wild-type *Cacna2d2* transcript is not detected in the brain and mutant transcripts would encode proteins that are unlikely functional.

In the 3 previously reported affected siblings mutated in *CACNA2D2*, the p.Leu1040Pro nonsynonymous change was associated with a clinical picture slightly different from the case described here: the three affected siblings had earlier onset of epileptic seizures (20–60 days of age vs. 5 months of the present case); they displayed atonic, clonic and tonic attacks without focality rather than partial or absence seizures; they did not show dysmorphic features; their EEG picture was consistent with a Lennox-Gastaut syndrome rather than being characterized by multifocal paroxysmal abnormalities and typical absences as observed in the present case. Epilepsy with different type of seizures, including partial, hemiclonic, and typical absence seizures seems to characterize the disruptive mutation described here. This suggests that only total loss of functional α2δ-2 may induce the distinctive epileptic features observed in the present case. Only studies involving larger numbers of patients with *CACNA2D2* mutations will provide confirmation of this hypothesis.

The presence of variants in *CELSR3* in two unrelated families with EE and *CACNA2D2* mutations could in principle be explained by a joint contribution of these two genes to the EE phenotype. Although *CELSR3*−/− mice display gross structural brain alterations that do not resemble any features of the affected children in the two families, [28] a role for the *CELSR3* mutations cannot be ruled out. Inactivation of *CELSR3* in mice causes abnormalities in cerebral cortex connectivity [29]. The two *CELSR3* mutations, p.Met2630Ile in the previously reported family and p.Gly2136Asp in the present one, are rare or novel nonsynonymous changes affecting highly conserved residues, and therefore are both variants with a putative detrimental role. However the present *CELSR3* mutation was not predicted as pathogenic by all the predictors used, unlike the one previously reported (**Table 1**) [11]. Therefore, in light of our results clearly showing the disruptive effect of the p.Asn432fs mutation, we explored the possibility that the *CELSR3* variants were only incidentally detected in the two families. Focusing on the analysis of the present single proband alone, we obtained that the detection of the p.Gly2136 Asp in *CELSR3* was not statistically significant. This is due to the relatively excessive length of its encoded protein (about 7-fold the genomic average), that inflates the number of variants that can be randomly drawn from this gene in a WES setting. Conversely, detection of p.Asn432fs in *CACNA2D2*, which encodes a shorter protein (about 2-fold the genomic average) resulted to be statistically relevant applying a stringent loss-of-function filtering. *CACNA2D2* and *CELSR3* are only 1.7 megabases apart on chromosome 3. It follows that a haplotype harboring a *CACNA2D2* disease-causing mutation can be found to coincidentally carry a *CELSR3* rare or novel polymorphism, and that due to recent parental relatedness both the two variants appear in the homozygous state. In order to prove with certainty that the *CELSR3* does not contribute to cause the EE pathogenesis, further patients should be recruited that do show mutations in *CACNA2D2* but not in *CELSR3*. However, it is often difficult to recruit additional patients or families with an ultra-rare disorder such as the one affecting the present proband. Here, we demonstrated that functional and statistical validation together can be valuable resource to collect evidence of causality or non-causality of reliable WES variant findings.

In conclusion, our results strengthened the association of *CACNA2D2* mutations to EE. The data we collected suggested that α2δ-2 genotype-phenotype correlation may depend on different levels of residual α2δ-2 activity. Our analyses indicated that the *CELSR3* variants were potentially deleterious but likely incidental findings, demonstrating that molecular and statistical reasoning can assist in discriminating the true disease-causing from the reliable but non causative candidate variants that emerge from the individual WES data.

## Supporting Information

Table S1 **Extended autozygous regions identified in the proband.**
(DOC)

Table S2 **Results of the variant prioritization by Exomiser.**
(XLS)

## References

[1] Tavyev Asher YJ, Scaglia F (2012) Molecular bases and clinical spectrum of early infantile epileptic encephalopathies. Eur J Med Genet 55: 299–306. 22548976 10.1016/j.ejmg.2012.04.002

[2] Kullmann DM, Waxman SG (2010) Neurological channelopathies: new insights into disease mechanisms and ion channel function. J Physiol 588: 1823–7. 20375141 10.1113/jphysiol.2010.190652 PMC2901970

[3] Bidaud I, Mezghrani A, Swayne LA, Monteil A, Lory P (2006) Voltaged-gated calcium channels in genetic diseases. Biochim Biophys Acta 1763: 1169–74. 17034879 10.1016/j.bbamcr.2006.08.049

[4] Imbrici P, Jaffe SL, Eunson LH, Davies NP, Herd C, et al (2004) Dysfunction of the brain calcium channel CaV2.1 in absence epilepsy and episodic ataxia. Brain 127: 2682–92. 15483044 10.1093/brain/awh301

[5] Walker D, De Waard M (1998) Subunit interaction sites in voltage-dependent Ca2_ channels: role in channel function. Trends Neurosci 21: 148–154. 9554724 10.1016/s0166-2236(97)01200-9

[6] Gong HC, Hang J, Kohler W, Li L, Su TZ (2001) Tissue-specific expression and gabapentin-binding properties of calcium channel _2_ subunit subtypes. J Membr Biol 184: 35–43. 11687876 10.1007/s00232-001-0072-7

[7] Barclay J, Balaguero N, Mione M, Ackerman SL, Letts VA, et al (2001) Ducky mouse phenotype of epilepsy and ataxia is associated with mutations in the *Cacna2d2* gene and decreased calcium channel current in cerebellar Purkinje cells. J Neurosci 21: 6095–6104. 11487633 10.1523/JNEUROSCI.21-16-06095.2001 PMC6763162

[8] Brodbeck J, Davies A, Courtney J-M, Meir A, Balaguero N, et al (2002) The ducky mutation in *Cacna2d2* results in altered Purkinje cell morphology and is associated with the expression of a truncated a2d-2 protein with abnormal function. J Biol Chem 277: 7684–7693. 11756448 10.1074/jbc.M109404200

[9] Hoppa MB, Lana B, Margas W, Dolphin AC, Ryan TA (2012) α2δ expression sets presynaptic calcium channel abundance and release probability. Nature 486: 122–5. 22678293 10.1038/nature11033 PMC3376018

[10] Gao B, Sekido Y, Maximov A, Saad M, Forgacs E, et al (2000) Functional properties of a new voltage-dependent calcium channel alpha(2)delta auxiliary subunit gene (*CACNA2D2*). J Biol Chem 275: 12237–12242. 10766861 10.1074/jbc.275.16.12237 PMC3484885

[11] Edvardson S, Oz S, Abulhijaa FA, Taher FB, Shaag A, et al (2013) Early infantile epileptic encephalopathy associated with a high voltage gated calcium channelopathy. J Med Genet 50: 118–123. 23339110 10.1136/jmedgenet-2012-101223

[12] Li H, Durbin R (2010) Fast and accurate long-read alignment with Burrows-Wheeler transform. Bioinformatics 26: 589–95. 20080505 10.1093/bioinformatics/btp698 PMC2828108

[13] DePristo MA, Banks E, Poplin R, Garimella KV, Maguire JR, et al (2011) A framework for variation discovery and genotyping using next-generation DNA sequencing data. Nat Genet 43: 491–498. 21478889 10.1038/ng.806 PMC3083463

[14] Li H, Handsaker B, Wysoker A, Fennell T, Ruan J, et al (2009) The Sequence Alignment/Map format SAMtools. Bioinformatics 25: 2078–9. 19505943 10.1093/bioinformatics/btp352 PMC2723002

[15] Pippucci T, Benelli M, Magi A, Martelli PL, Magini P, et al (2011) EX-HOM (EXome HOMozygosity): a proof of principle. Hum Hered 72: 45–53. 21849793 10.1159/000330164

[16] Robinson P, Köhler S, Oellrich A, Sanger Mouse Genetics Project, Wang K, et al. (2013) Improved exome prioritization of disease genes through cross species phenotype comparison. Genome Res doi:10.1101/gr.160325.113 10.1101/gr.160325.113 PMC3912424 24162188

[17] Schwarz JM, Rödelsperger C, Schuelke M, Seelow D (2010) MutationTaster evaluates disease-causing potential of sequence alterations. Nat Methods. 7: 575–6. 10.1038/nmeth0810-575 20676075

[18] Kumar P, Henikoff S, Ng PC (2009) Predicting the effects of coding non-synonymous variants on protein function using the SIFT algo- rithm. Nat Protoc 4: 1073–1081. 19561590 10.1038/nprot.2009.86

[19] Adzhubei IA, Schmidt S, Peshkin L, Ramensky VE, Gerasimova A, et al (2010) A method and server for predicting damaging missense mutations. Nat Methods 7: 248–249. 20354512 10.1038/nmeth0410-248 PMC2855889

[20] Smedley D, Oellrich A, Köhler S, Ruef B (2013) Sanger Mouse Genetics Project, et al (2013) PhenoDigm: analyzing curated annotations to associate animal models with human diseases. Database 9: bat025. 10.1093/database/bat025 PMC3649640 23660285

[21] Zhi D, Chen R (2012) Statistical Guidance for Experimental Design and Data Analysis of Mutation Detection in Rare Monogenic Mendelian Diseases by Exome Sequencing. PLoS ONE 7: e31358. 22348076 10.1371/journal.pone.0031358 PMC3277495

[22] Pennisi E (2012) “ENCODE Project Writes Eulogy For Junk DNA”. Science 337: 1159–1160. 22955811 10.1126/science.337.6099.1159

[23] International Human Genome Sequencing Consortium (2001) “Initial sequencing and analysis of the human genome”. Nature 409: 860–921. 11237011 10.1038/35057062

[24] Dubowitz V, Sewry CA (2007) Muscle biopsy: A practical approach. Sauders Elsevier, 2007, 3^rd^ ed.

[25] Bradford MM (1976) A rapid and sensitive method for the quantitation of microgram quantities of protein utilizing the principle of protein-dye binding. Anal Biochem 72: 248–54. 942051 10.1016/0003-2697(76)90527-3

[26] Schneider CA, Rasband WS, Eliceiri KW (2012) NIH Image to ImageJ: 25 years of image analysis. Nature Methods 9: 671–675. 22930834 10.1038/nmeth.2089 PMC5554542

[27] Uhlen M, Oksvold P, Fagerberg L, Lundberg E, Jonasson K, et al (2010) Towards a knowledge-based Human Protein Atlas. Nat Biotechnol 28: 1248–50. 21139605 10.1038/nbt1210-1248

[28] Tissir F, Bar I, Jossin Y, Goffinet AM (2005) Protocadherin *Celsr3* is crucial in axonal tract development. Nature Neurosci 8: 451–457. 15778712 10.1038/nn1428

[29] Zhou L, Bar I, Achouri Y, Campbell K, De Backer O, et al (2008) Early forebrain wiring: genetic dissection using conditional *Celsr3* mutant mice. Science 320: 946–949. 18487195 10.1126/science.1155244 PMC2746700

[30] Cooper GM, Stone EA, Asimenos G (2005) NISC Comparative Sequencing Program, Green ED, et al (2005) Distribution and intensity of constraint in mammalian genomic sequence. Genome Res 15: 901–13. 15965027 10.1101/gr.3577405 PMC1172034

