# Supplementary material for: A Novel Null Homozygous Mutation Confirms CACNA2D2 as a Gene Mutated in Epileptic Encephalopathy
Source: PLoS One. 2013 Dec 16;8(12):e82154. doi: 10.1371/journal.pone.0082154 (PMC3864908; doi:10.1371/journal.pone.0082154)
Supplement: Table S1 — Extended autozygous regions identified in the proband. (DOC) [file pone.0082154.s001.doc]

| Chromosome | 5’boundary(bp) | 3’boundary (bp) | 5’boundary (SNP) | 3’boundary (SNP) | Length (Mb) |
| --- | --- | --- | --- | --- | --- |
| chr2 | 232378961 | 243061183 | rs142490232 | rs188068415 | 10,7 |
| chr3 | 45677739 | 63825488 | rs146835695 | rs143457856 | 18,1 |
| chr5 | 106712639 | 112176196 | rs115264262 | rs137988845 | 5,5 |
| chr6 | 37300284 | 45515367 | rs2295243 | rs180855207 | 8,2 |
| chr6 | 49398896 | 57244672 | rs74854132 | rs5011404 | 7,8 |
| chr6 | 69345711 | 74537729 | rs141929159 | rs111445304 | 5,2 |
| chr10 | 12142306 | 18966952 | rs183780690 | rs147068091 | 6,8 |
| chr10 | 20105442 | 26878723 | rs7918855 | rs77429242 | 6,7 |
| chr17 | 25621173 | 30980907 | rs188344504 | rs149497642 | 5,4 |
| chr19 | 17767114 | 24346309 | rs150006719 | rs192946921 | 6,6 |
| chr19 | 32843652 | 44285461 | rs188238472 | rs117604122 | 11,4 |
| chr19 | 51013524 | 59095469 | rs188064442 | rs1133047 | 8,1 |
| chr20 | 45362377 | 52560043 | rs113496485 | rs77512516 | 7,2 |
